# Supplementary material for: Prevalence and Characterization of Beta-Lactam and Carbapenem-Resistant Bacteria Isolated from Organic Fresh Produce Retailed in Eastern Spain
Source: Antibiotics (Basel). 2023 Feb 14;12(2):387. doi: 10.3390/antibiotics12020387 (PMC9952115; doi:10.3390/antibiotics12020387)
Supplement: Supplementary file 1 [file antibiotics-12-00387-s001.zip › antibiotics-2161643-supplementary.pdf]

Supplementary Material

Table S1. Identification of isolates.

Isolate	Origin	API identification (>98,7%)	Identification by 16 rRNA gene partial sequencing	Homology	BLAST Accession number
IS1	Spinach	*Stenotrophomonas maltophilia*			
IS2	Lettuce	*Rahnella aquatilis*			
IS3	Spinach	*Pseudomonas oryzihabitans*			
IS4	Spinach	*Stenotrophomonas sp.*			
IS5	Cabbage	*Enterobacter cloacae* (95,4%)	*Enterobacter* sp. Cepa NOD9 16S ribosomal RNA gene, partial sequence	98,22%	gi|1391915696| MH392323.1
IS6	Cabbage	*Stenotrophomonas maltophilia* (96,7%)	*Stenotrophomonas maltophilia* cepa JM11 16S ribosomal RNA gene, partial sequence	100%	gi|1853410410|MT605300.1
IS7	Strawberry	*Pseudomonas putida*			
IS8	Spinach	*Stenotrophomonas sp.*			
IS9	Spinach	*Stenotrophomonas sp.*			
IS10	Lettuce	*Pseudomonas oryzihabitans*			
IS11	Cabbage	*Burkholderia cepacia*			
IS12	Lettuce	*Pseudomonas oryzihabitans*			
IS13	Spinach	*Acinetobacter baumannii* (5,6%)	*Pseudomona putida* cepa SG 5 16S ribosomal RNA gene, partial sequence	97,87%	gi|1158499428|KY750243.1
IS14	Spinach	*Pseudomonas putida*			
IS15	Spinach	*Enterobacter cloacae* (97,7%)	*Enterobacter hormaechei* cepa LS36 16S ribosomal RNA gene, partial sequence	100%	gi|1841784216|MT470964.1

IS16	Lettuce	*Proteus penneri*			
1S17	Spinach	*Acinetobacter baumanii*			
IS18	Spinach	*Stenotrophomonas maltophilia*			
IS19	Strawberry	*Pseudomonas luteola*			
IS20	Cabbage	*Enterobacter cloacae* (95,4%)	*Enterobacter ludwigii* cepa ESM19 16S ribosomal RNA gene, partial sequence	100%	gi|1702088840|MN173463.1
IS21	Spinach	*Elizabethkingia meningoseptica*			
IS22	Lettuce	*Stenotrophomonas maltophilia*			
IS23	Spinach	*Elizabethkingia meningoseptica*			
IS24	Spinach	*Stenotrophomonas sp.*			
IS25	Spinach	*Pseudomonas oryzihabitans*			
IS26	Spinach	*Serratia sp.*			
IS27	Lettuce	*Acinetobacter baumannii*			
IS28	Lettuce	*Stenotrophomonas maltophilia*			
IS29	Lettuce	*Stenotrophomonas spp.*			
IS30	Cabbage	*Salmonella ssp* (84,5%)//*Citrobacter freundii* (7,3%)	*Pseudomonas putida* cepa YC-AE1 16S ribosomal RNA gene, partial sequence	96,40%	gi|1540304109|MK318658.1
IS31	Cabbage	*Sphingebacterium multivorum*			
IS32	Cabbage	*Burkholderia cepacia*			
IS33	Cabbage	*Pseudomonas fluorescens*			
IS34	Cabbage	*Acinetobacter baumannii* (97,1%)	*Enterobacter cancerogenus* strain MPS1416S	100%	gi|1855725917|MT612440.1
IS35	Spinach	*Pseudomonas aeruginosa*			

IS36	Spinach	*Stenotrophomonas maltophilia*			
IS37	Spinach	*Stenotrophomonas sp.*			
IS38	Spinach	*Pasteurella pneumotropica*			
IS39	Spinach	*Pasteurella pneumotropica*			
IS40	Lettuce	*Stenotrophomonas maltophilia*			
IS41	Cabbage	*Acinetobacter baumannii* (52,1%)	*Pseudomonas* sp. DGC-2 16S ribosomal RNA gene, partial sequence	97,76%	gi|300675907|HM347049.1
IS42	Lettuce	*Stenotrophomonas maltophilia*			
IS43	Lettuce	*Stenotrophomonas sp.*			
IS44	Spinach	*Pasteurella pneumotropica*			
IS45	Spinach	*Stenotrophomonas sp.*			
IS46	Cabbage	*Stenotrophomonas sp.*			
IS47	Lettuce	*Pseudomonas putida*			
IS48	Spinach	*Rahnella aquatilis*			
IS49	Spinach	*Pseudomonas luteola*			
IS50	Spinach	*Enterobacter sp.*			
IS51	Lettuce	*Acinetobacter baumannii*			
IS52	Lettuce	*Stenotrophomonas maltophilia*			
IS53	Lettuce	*Acinetobacter baumannii*			
IS54	Spinach	*Stenotrophomonas sp.*			
IS55	Spinach	*Pseudomonas oryzihabitans*			

IS56	Spinach	*Acinetobacter baumannii*			
IS57	Spinach	*Pseudomonas oryzihabitans*			
IS58	Strawberry	*Klebsiella pneumoniae ssp neumoniae 2* (81'1%)	*Raoutella terrigena* cepa NCTC13098 genome assembly, chromosome: 1	99,81%	gi|1531751523|LR131271.1
IS59	Spinach	*Stenotrophomonas maltophilia*			
IS60	Spinach	*Stenotrophomonas maltophilia*			
IS61	Spinach	*Stenotrophomonas sp.*			
IS62	Spinach	*Klebsiella oxytoca* (16,3%)	*Pantoea ananatis* cepa FDAARGOS_680 chromosome, complete genome	99,63%	gi|1860091948|CP054912.1
IS63	Spinach	*Enterobacter cloacae*			
IS64	Spinach	*Enterobacter cloacae*			
IS65	Spinach	*Pseudomonas fluorescens*			
IS66	Lettuce	*Stenotrophomonas sp.*			
IS67	Lettuce	*Stenotrophomonas sp.*			
IS68	Lettuce	*Burkholderia cepacia*			
IS69	Lettuce	*Rahnella aquatilis*			
IS70	Lettuce	*Rahnella aquatilis*			
IS71	Lettuce	*Stenotrophomonas maltophilia*			
IS72	Lettuce	*Enterobacter cloacae*			
IS73	Spinach	*Rahnella aquatilis*			
IS74	Strawberry	*Serratia ficaria* (98,7%)	*Pseudomonas oryzihabitans* cepa 2.1 16S ribosomal RNA gene, partial sequence	100%	gi|1811132577|MT089709.1

IS75	Strawberry	*Serratia ficaria* (96'9%)	*Pseudomonas psychrotolerans* cepa CS51 chromosome, complete genome	99,55%	gi|1690455242|CP021645.1
IS76	Spinach	*Pantoea sp.*			
IS77	Spinach	*Serratia sp.*			
IS78	Spinach	*Buttiauxella agrestis*			
IS79	Cabbage	*Acinetobacter baumannii* (97,1%)	*Pseudomona reinekei* cepa SN8 16S ribosomal RNA gene, partial sequence	99,44%	gi|485477312|KC790310.1
IS80	Strawberry	*Pantoea*			
IS81	Lettuce	*Stenotrophomonas maltophilia*			
IS82	Lettuce	*Pantoea sp.*			
IS83	Spinach	*Leclercia adecarboxylata*			
IS84	Spinach	*Pantoea sp.*			
IS85	Lettuce	*Pseudomonas oryzihabitans*			
IS86	Spinach	*Sphingobacterium multivorum*			
IS87	Lettuce	*Acinetobacter baumannii*			
IS88	Lettuce	*Pseudomonas luteola*			
IS89	Spinach	*Acinetobacter baumannii*			
IS90	Spinach	*Burkholderia cepacia*			
IS91	Strawberry	*Klebsiella pneumoniae ssp neumoniae 2* (85'3%)	*Raoutella terrigena* cepa D56_MA5R 16S ribosomal RNA gene, partial sequence	100%	gi|1633436682|MK883162.1
IS92	Lettuce	*Pseudomonas putida*			
IS93	Spinach	*Pantoea sp.*			

IS94	Spinach	*Acinetobacter baumannii*			
IS95	Cabbage	*Pseudomonas fluorescens*			
IS96	Cabbage	*Enterobacter cloacae* (97,7%)	*Pseudomonas* sp. Cepa Str-1a 16S ribosomal RNA gene, partial sequence	99,74%	gi|1615643301|MK789741.1
IS97	Spinach	*Enterobacter cloacae* (97,7%)	*Enterobacter hormaechei* cepa LS36 16S ribosomal RNA gene, partial sequence	100%	gi|1841784216|MT470964.1
IS98	Spinach	*Acinetobacter baumannii* (52,1%)	*Acinetobacter baumannii* aislado BSA 40 16S ribosomal RNA gene, partial sequence	97,58%	gi|1622984671|MK824869.1
IS99	Cabbage	*Pseudomonas putida*			
IS100	Cabbage	*Stenotrophomonas sp.*	*Stenotrophomonas maltophilia* cepa UCD18.5 16S ribosomal RNA gene, partial sequence	100%	gi|1005392155|KU851240.1
IS101	Cabbage	*Acinetobacter baumannii* (52,1%)	*Acinetobacter calcoaceticus* cepa GDLB-46 16S ribosomal RNA gene, partial sequence	91,80%	gi|1616230209|MK791720.1
IS102	Cabbage	*Ralstonia pickettii*			
IS103	Spinach	*Pseudomonas putida*			
IS104	Spinach	*Pseudomonas putida*			
IS105	Cabbage	*Pseudomonas putida*			
IS106	Cabbage	*Pseudomonas putida*			
IS107	Cabbage	*Pseudomonas putida*			
IS108	Cabbage	*Pseudomonas putida*			
IS109	Cabbage	*Ralstonia pickettii*			
IS110	Cabbage	*Pseudomonas putida*			
IS111	Cabbage	*Pseudomonas putida*			

IS112	Cabbage	*Klebsiella pneumoniae ssp rhinoscleromantis or ozanae*	*Pseudomonas psyfhrotolerans* 16S ribosomal RNA gene, partial sequence	99,78%	gi|762217833|LN774322.1
IS113	Cabbage	*Pseudomonas fluorescens*			
IS114	Cabbage	*Pasteurella pneumotropica* (66,3%)	*Pseudomonas* sp. Cepa 102515 16S ribosomal RNA gene, partial sequence	99,68%	gi|1587660348|MK610450.1
IS115	Cabbage	*Acinetobacter baumannii* (5,6%)	*Pseudomona* sp. Cepa 102515 16S ribosomal RNA gene, partial sequence	99,35%	gi|1587660348|MK610450.1
IS116	Cabbage	*Klebsiella pneumoniae ssp rhinoscleromantis or ozanae*	*Pseudomonas oryzihabitans* cepa 2.1 16S ribosomal RNA gene, partial sequence	100%	gi|1811132577|MT089709.1
IS117	Strawberry	*Pseudomonas putida*			
IS118	Lettuce	*Pseudomonas putida*			
IS119	Lettuce	*Acinetobacter baumannii*			
IS120	Lettuce	*Burkholderia cepacia*			
IS121	Cabbage	*Pseudomonas putida Ralstonia pickettii* (89,7%)			
IS122	Cabbage	*Acinetobacter baumannii* (97'1%)	*Pseudomonas* sp. SWI7 chromosome, complete genome	72,38%	gi|1685755910|CP040930.1
IS123	Cabbage	*Serratia ficaria* (98'7%)	*Pseudomonas* sp. Cepa SK 09 16S ribosomal RNA gene, partial sequence	99,75%	gi|1128613278|KY427439.1
IS124	Cabbage	*Klebsiella pneumoniae ssp rhinoscleromatis* (89.6%)	*Pseudomonas* sp. Cepa HBUM200048 16S ribosomal RNA gene, partial sequence	97,74%	gi|1193729528|KY945802.1
IS125	Cabbage	*Serratia ficaria* (96'9%)	*Pseudomonas pyschrotolerans* cepa DHG28 16S ribosomal RNA gene, partial sequence	100%	gi|1784846536|MN833623.1
IS126	Strawberry	*Pseudomonas fluorescens*			
IS127	Spinach	*Pseudomonas aeruginosa*			
IS128	Cabbage	*Pseudomonasfliorescens*			

IS129	Cabbage	*Pseudomona luteola* (91,7%)//*Acinetobacter baumannii* (5,6%)	*Pseudomona* sp. Cepa HBUM206424 16S ribosomal RNA gene, partial sequence	99,51%	gi|1847283283|MT540518.1
IS130	Cabbage	*Stenotrophomonas maltophilia* (76'1%)	*Pseudomonas* sp. Cepa YL11 16S ribosomal RNA gene, partial sequence	99,80%	gi|1337931694|MG877640.1
IS131	Strawberry	*Stenotrophomonas maltophilia* (76'1%)	*Pseudomonas* sp. Cepa YL11 16S ribosomal RNA gene, partial sequence	99,80%	gi|1337931694|MG877640.1
IS132	Lettuce	*Pseudomonas putida*			
IS133	Lettuce	*Pseudomonas putida*			
IS134	Cabbage	*Pseudomonas putida*			
IS135	Cabbage	*Pseudomonas fluorescens*			
IS136	Cabbage	*Pseudomonas putida*			
IS137	Strawberry	*Serratia ficaria* (98,7%)	*Pseudomonas oryzihabitans* cepa 2.1 16S ribosomal RNA gene, partial sequence	100%	gi|1811132577|MT089709.1
IS138	Strawberry	*Acinetobacter baumannii*			
IS139	Strawberry	*Acinetobacter baumannii*			
IS140	Cabbage	*Acinetobacter baumannii* (5,6%)	*Pseudomonas reidholzensis* cepa D98_SO4R 16S ribosomal RNA gene, partial sequence	99,78%	gi|1633436717|MK883197.1
IS141	Cabbage	*Pseudomonas fluorescens*			
IS142	Cabbage	*Acinetobacter baumannii*			
IS143	Cabbage	*Pseudomonas putida*			
IS144	Cabbage	*Achromobacter xylosidans*			
IS145	Cabbage	*Pseudomonas fluorescens*			
IS146	Strawberry	*Klebsiella pneumoniae ssp neumoniae 2* (85'3%)	*Raoutella terrigena* cepa D56_MA5R 16S ribosomal RNA gene, partial sequence	99,81%	gi|1633436682|MK883162.1

IS147	Cabbage	*Pseudomonas putida*			
IS148	Cabbage	*Pseudomonas fluorescens*			
IS149	Cabbage	*Pseudomonas fluorescens*			
IS150	Cabbage	*Pseudomonas fluorescens*			
IS151	Strawberry	*Ochrobactrum anthropi*			
IS152	Strawberry	*Pseudomonas putida*			
IS153	Cabbage	*Pseudomonas fluorescens*			
IS154	Cabbage	*Acinetobacter baumannii* (52,1%)	*Acinetobacter calcoaceticus* cepa Sneb530 16S ribosomal RNA gene, partial sequence	100,00%	gi|1783758530|MN826157.1
IS155	Cabbage	*Pseudomonas fluorescens*			
IS156	Cabbage	*Stenotrophomonas maltophilia* (96,7%)	*Stenotrophomonas* sp. NTA 16S ribosomal RNA gene, partial sequence	99,36%	gi|1622984744|MK824942.1
IS157	Cabbage	*Pseudomonas putida*			
IS158	Cabbage	*Pseudomonas putida*			
IS159	Cabbage	*Pseudomonas putida*			
IS160	Strawberry	*Pseudomonas putida*			
IS161	Cabbage	*Yersinia enterococolítica* (97'9%)	*Enterobacter* sp. Cepa MD10F 16S ribosomal RNA gene, partial sequence	100%	gi|1680430374|MN049924.1
